# Modulation of Chromatin Remodelling Induced by the Freshwater Cyanotoxin Cylindrospermopsin in Human Intestinal Caco-2 Cells

**DOI:** 10.1371/journal.pone.0099121

**Published:** 2014-06-12

**Authors:** Antoine Huguet, Aurélie Hatton, Romain Villot, Hélène Quenault, Yannick Blanchard, Valérie Fessard

**Affiliations:** 1 Contaminant Toxicology Unit, Fougères Laboratory, Anses, Fougères Cedex, France; 2 Viral Genetics and Bio-security Unit, Ploufragan-Plouzané Laboratory, Anses, Site des Croix, Ploufragan, France; St. Georges University of London, United Kingdom

## Abstract

Cylindrospermopsin (CYN) is a cyanotoxin that has been recognised as an emerging potential public health risk. Although CYN toxicity has been demonstrated, the mechanisms involved have not been fully characterised. To identify some key pathways related to this toxicity, we studied the transcriptomic profile of human intestinal Caco-2 cells exposed to a sub-toxic concentration of CYN (1.6 µM for 24hrs) using a non-targeted approach. CYN was shown to modulate different biological functions which were related to growth arrest (with down-regulation of *cdkn1a* and *uhrf1* genes), and DNA recombination and repair (with up-regulation of *aptx* and *pms2* genes). Our main results reported an increased expression of some histone-modifying enzymes (histone acetyl and methyltransferases MYST1, KAT5 and EHMT2) involved in chromatin remodelling, which is essential for initiating transcription. We also detected greater levels of acetylated histone H2A (Lys5) and dimethylated histone H3 (Lys4), two products of these enzymes. In conclusion, CYN overexpressed proteins involved in DNA damage repair and transcription, including modifications of nucleosomal histones. Our results highlighted some new cell processes induced by CYN.

## Introduction

The toxin cylindrospermopsin (CYN) is produced by several freshwater cyanobacteria species such as *Cylindrospermopsis raciborskii*, which is nowadays common throughout the world, as its recent occurrence in temperate regions attests [1], [2]. CYN is a 415 Da water soluble tricyclic alkaloid containing a guanidinium group combined with hydroxymethyluracil [3], [4]. Unlike other cyanobacterial toxins which are generally sequestered inside cyanobacteria until death, CYN can be released in water during blooms (up to 90% of the CYN produced) [5], [6]. The major route of human exposure to this toxin is oral through drinking water and also probably through contaminated food by bioaccumulation [1], [7]. Several cases of human intoxications due to CYN water contamination have been reported in Australia and Brazil [8], [9]. These events revealed that CYN may induce various injuries, including gut damage, occasionally leading to death. Therefore, this cyanobacterial toxin has been recognised as a potential public health risk in several countries [10].

CYN has been described *in vitro* to induce cytotoxicity in different cell models including human cell lines from the intestine and liver, the main target organs of the toxin [11]–[16]. The cyanotoxin has been demonstrated to act through an irreversible inhibition of eukaryotic protein synthesis in *in vitro* experiments using cellular components [17], [18] or cell lines [19]–[21]. However, CYN cytotoxicity was not solely due to protein synthesis inhibition, and other CYN-induced effects probably contributed to the toxic process [22]. Indeed, the inhibition of glutathione (GSH) synthesis led to a decrease in GSH content, and consequently to an increase of reactive oxygen species and DNA damage [15], [20], [23]–[26]. However, protein synthesis inhibition occurred in metabolism-incompetent cells, as well as in metabolism-competent models treated with cytochrome P450 inhibitors [22], [27], while GSH decrease and cytotoxicity were limited by cytochrome P450 inhibitor treatment [27]–[30].

Due to the various effects induced by CYN and contributing to the toxic process, and considering that oral intake is the major route of human exposure to CYN, and that the intestine is one of the target organs, we investigated the cellular and molecular mechanisms of action involved in CYN toxicity on differentiated human intestinal Caco-2 cells. This cellular monolayer is a relevant *in vitro* model exhibiting functional and morphological characteristics similar to enterocytes [31]. For this purpose, we studied the transcriptomic profile of differentiated Caco-2 cells exposed to a sub-toxic concentration of CYN after 24h exposure using a non-targeted approach with microarrays. When pathways were identified, gene expression and the functions of key biomarkers were evaluated.

## Materials and Methods

### Chemicals

Cell culture products were purchased from Gibco (Invitrogen, Cergy Pontoise, France). CYN was kindly provided by Dr A. Humpage (Australian Water Quality Center, Adelaide, Australia) and prepared in sterile water. The “In cytotox” kit was supplied by Biogenic (Perols, France).

### Cell culture and differentiation

Caco-2 cells were obtained from the American Type Culture Collection (ATCC HTB-37, LGC Standards, Molsheim, France) and used at passages 30–34. Cells were grown in Minimum Essential Medium containing 1 g/L glucose, Earle's salts and L-glutamine (MEM GlutaMAX), supplemented with 1% non-essential amino acids, 50 IU/mL penicillin, 50 µg/mL streptomycin and 20% foetal calf serum (FCS) at 37°C in an atmosphere containing 5% CO_2_. Cells were seeded in 75 cm^2^ culture flasks and passaged twice a week when confluence was reached. For differentiation, Caco-2 cells were seeded at 6×10^4^ cells/cm^2^ in 48-well plates for the cytotoxicity assay, or 24-well plates for microarray experiments, or Nunc Edge 96-well Thin Bottom microplates (Thermo Fisher Scientific) for immunolabelling experiments, and grown in medium supplemented with 10% FCS for 23–25 days. The medium was changed three times a week.

### Toxin exposure

Regarding cytotoxicity data of the literature mentioned in the introduction, we chose for the cytotoxicity assay a concentration range of CYN that frame the sub-toxic concentration which will be used for the later experiments. In this way, differentiated Caco-2 cells were exposed for 24 hrs to a range of 9 concentrations (from 0.2 to 50 µM CYN) in fresh FCS-free medium for the cytotoxicity assay, or to 1.6 µM of CYN for the microarray and immunolabelling experiments. A vehicle control (sterile water) was also included for each experiment. For the cytotoxicity assay, three independent experiments (biological replicates) were performed, each including three technical replicates (wells) per treatment condition. For the microarray and immunolabelling assays, six independent experiments were performed; for the immunolabelling assay, three technical replicates (wells) per treatment condition were included for each independent experiment.

### Cytotoxicity

After 24 hrs of treatment with CYN, a neutral red uptake assay was performed with the “In cytotox” kit according to the manufacturer's instructions (Xenometrix, Allschwil, Switzerland). Absorbance was measured with a microplate reading spectrofluorometer (FLUOstar OPTIMA, BMG Labtech, Champigny-sur-Marne, France). For each independent experiment, the median of the technical replicates was expressed relative to that of the vehicle control. These relative medians were used for statistical analyses.

### Total RNA extraction

Following cell treatments, total RNA was isolated using the NucleoSpin RNA II kit according to the manufacturer's instructions with a final elution volume of 10 µL of RNase free water (Macherey-Nagel, Hoerd, France). RNA was quantified with the BioSpec-Nano (Shimadzu, Marne la Vallée, France), and RNA integrity was assessed with the “Experion RNA StdSens analysis” kit using the Experion automated electrophoresis system (Bio-Rad, Marnes-la-Coquette, France). Only RNA with an RNA quality indicator ≥ 9 was used for further experiments (Experion software 3.0; Bio-Rad). A negative extraction control of lysis buffer RA1 was included for contamination assessment.

### Microarray

For probe preparation, 200 ng of total RNA were reverse transcribed into double strand cDNA and then amplified and labelled into cRNA with cyanine-3 CTP using the “Low Input QuickAmp Labeling” kit according to the manufacturer's protocol (Agilent Technologies, Massy, France). Cyanine-3-labeled cRNA was purified using the “RNeasy Mini” kit according to the manufacturer's instructions (Qiagen, Courtaboeuf, France), cyanine incorporation was monitored with a NanoPhotometer (Implen, Munich, Germany), and cyanine-3-labeled cRNA was fragmented for 30 min at 60°C. For the experiments we used 4 X 44K Whole Human Genome 70-mer oligo-chips (G4112F; Agilent Technologies). Hybridisation of Cyanine-3-labeled cRNA samples was performed onto each microarray for 17 hrs at 65°C with an HSPro4800 hybridisation automatic station (Tecan) and using the Gene Expression Wash Pack kit (Agilent Technologies) and the “Stabilization and Drying Solution” kit (Agilent Technologies) according to the manufacturers' protocols. Thereafter, the absorbance was measured at 532 nm with an Innoscan 900AL scanner (Innopsys, Carbonne, France).

### Data analysis

Raw data, corresponding to the median pixel intensity for each probe, were extracted with Mapix software, version 5.0. The quality of the extracted raw data was checked for signal homogeneity using the Pearson correlation. A Lowess normalisation was performed on the raw data with R software using the Linear Model for Microarray Data Bioconductor package [32]. A background signal was calculated from the mean of the one hundred lowest values of each sample. Following this, the normalised data were filtered on threshold intensity (3 times the background signal): for each probe, values were selected if the median value for at least one experimental condition was higher than the threshold intensity. This dataset, including the 28 616 post-filtering probes, was labelled “filtered data”. These data have been deposited in NCBI's Gene Expression Omnibus and are accessible through GEO Series accession number GSE55723.

From the “filtered data”, the differentially expressed genes were selected at *P*<0.05 (Student t-test) and with a fold change (FC) greater than 2 (for “up-regulated genes”), or less than 0.5 (for “down-regulated genes”) leading to two distinct clusters. In order to identify over-represented gene categories, analysis was performed on these clusters using the GoMiner application (http://www.discover.nci.nih.gov/gominer/index.jsp) which is based on the gene ontology (GO) annotation system. GO terms with a false discovery rate (FDR) score lower than 0.05 and an enrichment score greater than 1.5 was declared to be significant.

Significance analysis of microarrays (SAM) method was also performed on the “filtered data” with R software and the samr plug-in. The set of differentially expressed genes after cell exposure to CYN was identified by a two-class unpaired analysis with an FDR lower than 0.01. Biological mechanisms, pathways and functions corresponding to the set of genes resulting from the SAM analysis were identified with ingenuity pathways analysis (IPA) software (http://www.ingenuity.com).

### RT-qPCR

The minimum information for publication of quantitative real-time PCR experiments guidelines for qPCR assay design and reporting [33] were applied. Reverse transcription (RT) was performed with 500 ng of total RNA using the High Capacity RNA-to-cDNA kit (Applied Biosystems, Foster City, CA) according to the manufacturer's instructions. Reaction volume was set to 20 µL and RT was performed at 37°C for 60 min prior to a stopping step for 5 min at 95°C. Negative RT control of RNase-free water and a no-reverse transcription control (replacement of reverse transcriptase by RNase-free water) were included for assessing respectively any external contamination and the absence of DNA occurring during RNA extraction. The produced cDNAs were stored at −20°C.

The sequences of target genes were obtained from the National Center for Biotechnology Information GenBank sequence database (http://www.ncbi.nlm.nih.gov/). Primers were designed with OligoPerfect Designer (http://www.invitrogen.com). For each gene, at least one primer was designed on the exon-exon junction. *In silico* analysis of primer specificity was performed using nucleotide Basic Local Alignment Search Tool (http://www.ncbi.nlm.nih.gov/BLAST/). All primers were purchased from Sigma-Aldrich (Lyon, France). The gene *RPLP0* was chosen as a reference gene since it did not exhibit any significant variation in expression among the samples. Additional information on target genes and oligonucleotide primers are listed in Table S1.

Quantitative PCR was performed on a Chromo4Real-Time Detector in low-profile 8-white tubes strips (Bio-Rad). SYBR Green chemistry was used. Reactions were performed on three technical replicates in a total volume of 10 µl containing 1X Power SYBR GREEN PCR Master Mix (Applied Biosystems, Foster City, CA), 300 nM each primer, and 0.25 ng cDNA. Negative quantitative PCR controls of RNase-free water were included in each run for contamination assessment. The thermal cycling conditions were 94°C for 7 min, followed by 40 cycles of denaturation at 95°C for 15 s, annealing at the determined temperature for 15 s, and polymerisation at 72°C for 15 s. Opticon Monitor software (version 3.0; Bio-Rad) was used for the quantitative analysis, and melting curve analysis was used to check the specificity of each amplicon. Threshold C_q_s were calculated from a baseline subtracted curve fit. Calibration curves were established for each gene from a serial two-fold dilution of a reference sample (pool of cDNA samples). Using these calibration curves, for each sample, median relative amounts of mRNA of the target genes were calculated and then normalised to that of the reference gene, *RPLP0*. These normalised medians were used for statistical analyses and values were presented as arbitrary units.

### Immunolabelling

Following treatment, cells were fixed for 10 min with 4% paraformaldehyde in PBS, and permeabilised for 10 min with 0.2% Triton X-100 in PBS. Then, cells were incubated for 30 min with 0.05% Tween/1% BSA in PBS filtered at 0.22 µm (blocking solution), and successively incubated at room temperature for 2 hrs and 1 hr with, respectively, primary and secondary antibodies prepared in blocking solution. Primary antibodies: rabbit polyclonal Tip60/KAT5 (4 µg/mL; Millipore, 07-038), rabbit polyclonal EHMT2/G9a (1 µg/mL; Millipore, 07-551), mouse monoclonal MYST1 (2 µg/mL; Thermo Fisher Scientific, MA5-15345), rabbit polyclonal trimethyl-histone H3 (Lys9) (1 µg/mL; Millipore, 07-442), rabbit polyclonal dimethyl-histone H3 (Lys4) (0.5 µg/mL; Millipore, ABE250), rabbit polyclonal acetyl-histone H2A (Lys5) (0.5 µg/mL; Millipore, 07-290), rabbit monoclonal acetyl-histone H4 (Lys5) (0.3 µg/mL; Millipore, 04-118). Secondary antibodies: goat anti-rabbit IgG (H+L) (DyLight 488) (1 µg/mL; Thermo Fisher Scientific, 35552), goat anti-mouse IgG (H+L) (1 µg/mL; DyLight 550) (Thermo Fisher Scientific, 84540). For nuclear identification, cells were incubated for 5 min with DAPI 1 µg/mL/0.05% Tween in PBS filtered at 0.22 µm. Plates were scanned with a Thermo Scientific Arrayscan VTI HCS Reader (Thermo Fisher Scientific) and the generated images were analysed using the Spot Detector BioApplication (Thermo Fisher Scientific). For each independent experiment, 10 fields (size of each field: 660×660 µm) were analysed per well, and the spot average intensity was determined for each field. Thereafter the mean from the 30 spot average intensity (3 wells, 10 fields per well) was calculated. These means were used for statistical analyses and values were presented as arbitrary units.

### Statistical analysis

Statistical analyses were performed using GraphPad Prism software (version 5.0; GraphPad Software Inc., La Jolla, CA). For the cytotoxicity assay, data were analysed using the One-sample t test with “100” as the theoretical mean. Means were declared significantly different from 100 at *P*<0.05, and tendencies (*P*<0.1) were included. For the qPCR assay, for each treatment condition the variances were firstly compared using the Fisher test; when *P* > 0.1, data were analysed using the unpaired Student *t* test, and when *P*<0.1, data were analysed using the unpaired Student *t* test with Wlech's correction. Differences were declared significant at *P*<0.05. For immunolabelling, data were analysed using the paired Student *t* test. Differences were declared significant at *P*<0.05.The values presented were means ± SE.

## Results

### Cytotoxicity

**Figure 1:**
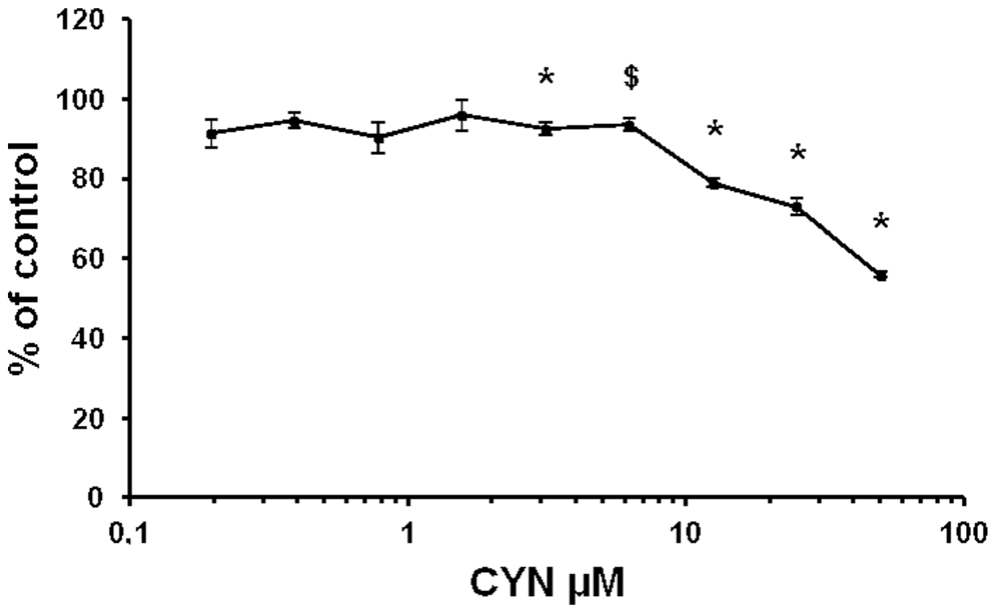
Cytotoxic effects of CYN in differentiated Caco-2 cells treated for 24 hrs. Cytotoxicity effect was measured by neutral red uptake assay. Values are presented as means ± SE, and expressed as percentages of the vehicle control. Three independent experiments were performed. *, $: values are (*, *P*<0.05) or tended ($, *P*<0.1) to be different from 100.

Significant cytotoxic effects of CYN were observed in differentiated Caco-2 cells after 24 hrs with a concentration-dependent decrease of viability (Figure 1). No significant differences were noticed for concentrations ranging from 0.2 to 1.6 µM CYN. However, 3.1 µM CYN induced a slight decrease in neutral red uptake, and cytotoxicity increased from 21% to 44% with 12.5 and 50 µM CYN respectively (*P*<0.05).

### Gene expression profiles

According to the cytotoxicity curve, a sub-toxic concentration of 1.6 µM CYN for differentiated Caco-2 cells (inducing no significant alteration of cell viability) was selected for microarrays and immunolabelling experiments. Among the 12 RNA extracts, only 11 samples (5 controls and 6 CYN) had an RNA quality indicator ≥ 9 and were used for further experiments. From the “filtered data”, considering an FC greater than 2 or less than 0.5, and a *P* - value <0.05, we identified a set of 572 genes with a differential expression between CYN treatment and control samples. The full list of these genes is given in Table S2. Among this set of genes, 522 were up-regulated and 50 were down-regulated. The two lists of genes were used to address the biological and molecular processes affected by CYN using the GoMiner application.

**Figure 2:**
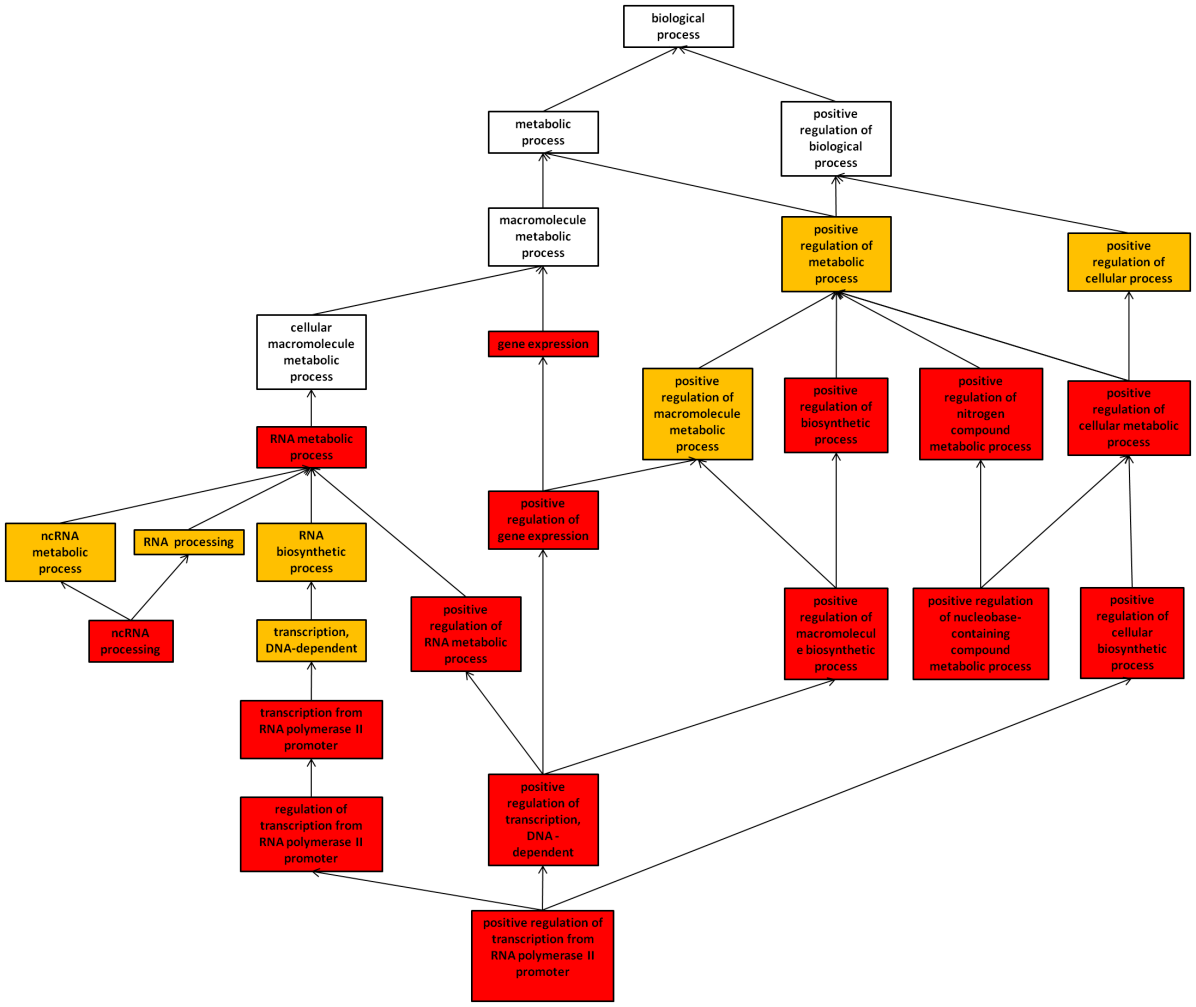
Tree of the biological processes up-regulated in differentiated Caco-2 cells after CYN exposure. The 522 genes showing up-regulation after 24 hrs exposure to 1.6 µM CYN were annotated within 22 biological processes (GO terms) with an enrichment score greater than 1.5. GO terms had a false discovery rate score of between 0.05 and 0.01 (in orange) or less than 0.01 (in red); white GO terms were ancestors.

From the 522 up-regulated genes, 22 biological processes (GO terms) were significantly enriched in our gene set, and are presented in a graph indicating their inter-relationships (Figure 2). Among the 22 GO terms, 7 had an FDR score between 0.05 and 0.01, while the 15 others had an FDR score lower than 0.01. The majority of these GO terms were related to transcription and biosynthesis of macromolecules such as RNA. Therefore, genes from the up-regulated cluster encoded mainly proteins implied in gene expression events. We also identified 11 significant molecular functions related to transcription and RNA modification events (Table S3). Considering cellular components, 12 GO terms mainly related to the nucleus and ribosome were significantly affected (Table S3). Our data suggest that proteins encoded by the 522 up-regulated genes are implied in both the regulation of transcription and post-transcription taking place respectively inside the nucleus and in the ribosomal machinery. The 50 down-regulated genes gave another list of enriched GO terms from which only 3 biological processes (“response to calcium ion”, “response to metal ion”, “response to inorganic ion”) and 1 cellular component (“membrane-bounded vesicle”) were identified, no molecular function being enriched (data not shown). However, no concordance between these GO terms was found.

**Figure 3:**
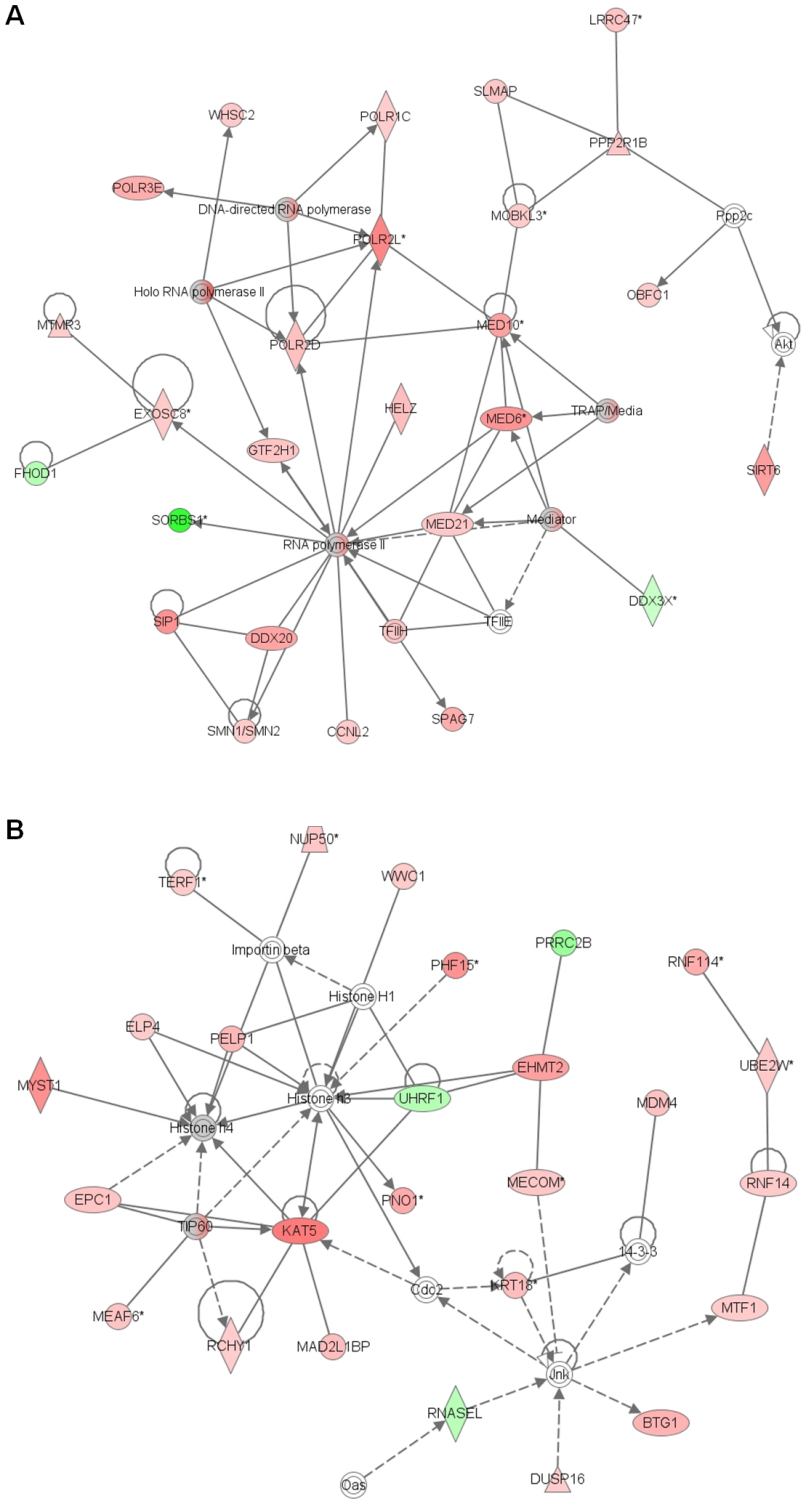
Associated network functions in differentiated Caco-2 cells after 24 hrs exposure to 1.6 µM CYN. The 2911 genes showing differential regulation were associated within networks using Ingenuity Pathway Analysis. In this figure the two networks with the highest score are presented. They are respectively associated with gene expression and RNA Post-Transcriptional Modification (A), or with gene expression (B) biological functions. Lines between gene products represent known interactions, with solid lines representing direct interactions and dashed lines representing indirect interactions. Genes showing up-regulation of expression levels in response to CYN exposure are in red, while down-regulated genes are in green.

With the SAM method on the “filtered data” and considering an FDR lower than 0.01, we identified a set of 2911 differentially expressed genes between CYN treated and control samples. Among these genes, 1711 had an FC greater than 1, and 1200 had an FC less than 1. This set of genes was used to identify biological functions affected by CYN exposure using IPA software. Firstly, we focused on the biological functions considering a *P*-value of less than 0.01, with at least 10 molecules involved. We identified several biological functions grouped into three major categories: gene expression, RNA post-transcriptional modifications, and DNA replication, recombination and repair (Table S4). Biological functions such as transcription of DNA endogenous promoter, processing of RNA, and modification of RNA, which are included in the first and second categories, were already identified with the GoMiner application. However, additional biological functions (DNA damage response of cells, repair of DNA, and modification of DNA), which include genes coding for proteins implied in the regulation of chromatin modifications, were affected. The analysis of the network functions revealed that four networks among the six having the highest score, were related to the three major categories previously mentioned. The first network described the relationships between gene products implied in transcription and post-transcriptional modification of RNA (Figure 3A): enzymes forming the RNA polymerase II complex such as POLR2D, POLR2L, POLR3E and POLR1C, transcription co-activation factors such as MED6, MED10, and MED21, and finally enzymes implied in RNA maturation. The second network identified gene products, such as acetyl transferases (MYST1, KAT5) or methyl transferase (EHMT2), involved in chromatin modification and particularly histone proteins post-translation modification (Figure 3B). These data suggested that exposure to CYN resulted in the modification of gene expression for proteins implied in transcriptional and post-transcriptional regulations, and in epigenetic events.

### Validation of CYN-induced gene expression

The expression level of some key genes from the two previously mentioned networks (transcription and post-transcriptional modification of RNA, and histone proteins post-translation modification) was evaluated by RT-qPCR. The specificity of primers using nucleotide Basic Local Alignment Search Tool and the melting curve was checked for each target gene. For contamination assessment, results revealed that ΔCqs of samples were at least 7 as compared to the different controls (extraction, RT, no-reverse transcription, and quantitative PCR). Results of the relative expression of the target genes are summarised in Table 1. In the first network, expression levels of *polr2d* and *polr2l* in CYN-treated Caco-2 cells were on average respectively 1.8 and 1.6-fold greater than the control (*P*<0.005). The increase in gene expression even reached 2.2 and 2.3-fold greater (*P*<0.005) for *med6* and *ddx20*. In the second network, the expression levels for *kat5*, *myst1* and *ehmt2* were respectively 2.2, 1.5 and 1.6-fold greater than the control (*P*<0.005).

**Table 1:** Relative gene expression of *polr2d*, *polr2l*, *med6*, *ddx20*, *kat5*, *myst1* and *ehmt2* in differentiated Caco-2 cells after 24 hrs exposure to 1.6 µM CYN.

Gene	Control	CYN treated	*P*-value
*polr2d*	1.25±0.08	2.22±0.15^*^	0.001
*polr2l*	0.66±0.04	1.05±0.06^*^	0.002
*med6*	0.57±0.01	1.24±0.07^**^	< 0.001
*ddx20*	1.10±0.04	2.49±0.27^*^	0.004
*kat5*	1.20±0.05	2.70±0.24^*^	0.002
*myst1*	1.21±0.03	1.83±0.06^**^	< 0.001
*ehmt2*	2.85±0.07	4.58±0.21^**^	< 0.001

Values are presented as means ± SE, and normalised to the reference gene *RPLP0*. Six independent experiments were performed.

^*, **^: significantly different from the control group (respectively *P*<0.005 and *P*<0.001).

### Evaluation of target protein levels

The levels of KAT5, MYST1, and EHMT2, three proteins involved in modification of the chromatin were also quantified by immunolabelling and image analysis. Results are summarised in table 2. The increase observed at the RNA level was confirmed at the protein level in diffferentiated Caco-2 cells exposed to 1.6 µM CYN for 24 h with on average +16%,+16%, and +24% (*P*<0.005) respectively for KAT5, MYST1, and EHMT2. As histone proteins are the targets of these enzymes, acetylation of histones H2A and H4 on Lys5, due to acetyl transferases KAT5 and MYST1, was evaluated. Compared to the control, the levels of acetyl-histone H2A (Lys5) was 13% greater (*P*<0.01), but no significant effect was detected for acetyl-histone H4 (Lys5). Methylation on Lys4 and Lys9 of histone H3 reflects activity of EHMT2, and was therefore evaluated. While the trimethyl-histone H3 (Lys9) levels was not affected by CYN treatment, the level of dimethyl-histone H3 (Lys4) increased by 14% (*P*<0.005) compared to the control.

**Table 2:** Levels of KAT5, MYST1, EHMT2, trimethyl-histone H3 (Lys9), dimethyl-histone H3 (Lys4), acetyl-histone H2A (Lys5) and acetyl-histone H4 (Lys5) in differentiated Caco-2 cells after 24 hrs exposure to 1.6 µM CYN.

Protein	Control	CYN treated	*P*-value
KAT5	114±15	132±9^*^	0.022
MYST1	77±16	89±17^**^	0.002
EHMT2	126±14	157±13^*^	0.015
trimethyl-histone H3 (Lys9)	129±14	127±10	>0.100
dimethyl-histone H3 (Lys4)	184±19	210±16^**^	0.003
acetyl-histone H2A (Lys5)	203±13	230±10^**^	0.005
acetyl-histone H4 (Lys5)	285±37	315±29	>0.100

Values are presented as means ± SE. For each sample, the levels of the target protein were evaluated by immunolabelling and image analysis. Six independent experiments were performed.

^*, **^: significantly different from the control group (respectively *P*<0.05 and *P*<0.005).

## Discussion

Prior to study the transcriptomic profile of differentiated Caco-2 cells exposed to CYN, a sub-toxic concentration of CYN after 24h of exposure was determined. Results revealed a concentration-dependant decrease of viability in CYN-exposed Caco-2 cells. Previous studies also reported a concentration-dependent decrease in cell viability on the same cell model with IC_50_ ranging from 5 to 50 µM probably due to the conditions used for the cytotoxicity test and if cells were differentiated or not [12], [13], [28]. In order to avoid any modulation of gene expression due to non-specific effects [34], [35], a sub-toxic concentration of CYN (1.6 µM), corresponding to the highest dose without any significant decrease in cell viability, was chosen.

To detect specific mechanisms involved in CYN toxicity at a molecular level, a non-targeted approach using pangenomic microarrays is appropriate. The GoMiner application and IPA software were used respectively for determine biological and molecular processes affected by CYN, and indentify key genes involved in these processes. We first observed that CYN modulated biological functions related to DNA recombination and repair. Experiments conducted previously *in vitro* as well as *in vivo* demonstrated that CYN induced DNA damage [26], [28], [36]–[40]. Using a targeted approach in human lymphocytes and HepG2 cells, the expression of some genes associated with growth arrest (*GADD45A*, *CDKN1A*, *UHRF1*) and DNA repair processes (*ERCC4*, *RAD51* and *BRCA2*) was modified following CYN exposure [40]–[42]. Although the expressions of *GADD45A*, *ERCC4*, *RAD51* and *BRCA2* were not significantly modulated in our experiment, data revealed down-regulation of *CDKN1A* and *UHRF1*, and expression modulation of other genes coding for proteins involved in damaged DNA repair, such as *APTX* and *PMS2* (up-regulated), and *BRCA1* (down-regulated). Therefore, CYN-induced DNA damage clearly activated the DNA repair machinery.

Our main results suggested that CYN induced an up-regulation of genes coding for proteins involved in the activation of transcriptional and post-transcriptional events. Our finding was confirmed when investigating some specific genes devoted to these processes. Indeed, the expression levels of key enzymes forming the RNA polymerase II complex, POLR2D and POLR2L, as well as *MED6*, a sub-unit of the co-activator complex Mediator, which are all required for gene transcription [43], were increased. Similar results were also obtained with another gene involved in RNA maturation, *DDX20* (coding for a member of the DEAD-box family of RNA helicases) [44]. Recruitment of co-activators acting on chromatin remodelling, including nucleosomal histone modifications, is also required for controlling transcription [45], [46]. Active or repressed chromatin is partially regulated through post-translational modifications (acetylation, methylation) of histone proteins, with the balance between the acetylated and methylated state of histones regulating key processes such as transcription, repair, recombination, and replication [47], [48]. Indeed, acetylation of both histones H4 and H2A on lysine 5 by the respective acetyltransferases MYST1 and KAT5 and dimethylation of histone H3 on Lys 4 by the methyltransferase EHMT2 have been shown to be essential for chromatin remodelling in order to initiate transcription in eukaryotes [49]–[52]. Therefore, both mRNA and protein levels of *MYST1, KAT5* and *EHMT2* enzymes were evaluated following CYN treatment. The expression of *KAT5* and the levels of acetylated histone H2A (Lys5) were increased. However, although the expression of *MYST1* was increased, the level of acetylated histone H4 (Lys5) was not affected by CYN. We also observed an increased expression level of *EHMT2* and higher levels of dimethylated histone H3 (Lys4). However, the trimethylation of H3 on lysine 9, a marker of repressed transcription [53], was not affected by CYN treatment. The histone modifications observed in our study confirm that DNA repair and transcription were activated following CYN exposure, and also probably following DNA damage induction.

In conclusion, differentiated Caco-2 cells exposed for 24 h to a sub-toxic concentration of CYN over-expressed the gene products involved in transcription and DNA damage repair, including modifications of nucleosomal histones. To our knowledge, this is the first study describing the effects on chromatin remodelling events after CYN exposure which were outlined by transcriptomic profiles. Although further investigations are needed, these data contribute to increasing knowledge of the various effects induced by CYN.

## Supporting Information

Table S1 **Target genes and oligonucleotide forward (F) and reverse (R) primers used in this study.**
(DOC)

Table S2 **List of the 522 up-regulated and 50 down-regulated genes in differentiated Caco-2 cells after 24 hrs exposure to 1.6 µM CYN.**
(DOC)

Table S3 **Cellular components and molecular functions for up-regulated genes in differentiated Caco-2 cells after 24 hrs exposure to 1.6 µM CYN.**
(DOC)

Table S4 **Categories and biological functions for differentially regulated genes in differentiated Caco-2 cells after 24 hrs exposure to 1.6 µM CYN.**
(DOC)
