# Supplementary material for: Modulation of Chromatin Remodelling Induced by the Freshwater Cyanotoxin Cylindrospermopsin in Human Intestinal Caco-2 Cells
Source: PLoS One. 2014 Jun 12;9(6):e99121. doi: 10.1371/journal.pone.0099121 (PMC4055761; doi:10.1371/journal.pone.0099121)
Supplement: Table S1 — Target genes and oligonucleotide forward (F) and reverse (R) primers used in this study. (DOC) [file pone.0099121.s001.doc]

**Table S1**. Target genes and oligonucleotide forward (F) and reverse (R) primers used in this study.

| **Gene symbol** | **Accession number** | **Target** | **Amplicon length (bp)** | **Sequence 5’ to 3’** | **Annealing T (C°)** |
| --- | --- | --- | --- | --- | --- |
| *polr2d* | NM_004805.3 |  | 129 | F : AGCAGAATGAGAGTGCAGAG | 56 |
|  |  |  |  | R : GGAGTAGCAAGCTACGAACA |  |
| *polr2l* | NM_021128.4 |  | 104 | F : GCCATGATCATCCCTGTACG | 62 |
|  |  |  |  | R : GCATCCCCCTCGGTGTACT |  |
| *ddx20* | NM_007204.4 |  | 171 | F : GCACAGCAGAGCACAACATT | 61 |
|  |  |  |  | R : GCATCAATCCCACGAGAAGT |  |
| *med6* | NM_005466.2 |  | 109 | F : GCCAAAAGGAAAGAAGAACC | 58 |
|  |  |  |  | R : CAGGCTTTAGCTGCACAAAT |  |
| *kat5* | NM_182710.2 | variant 1 | 181 | F : CATTTGACCAAGTGTGACCT | 56 |
|  |  |  |  | R : AGAAGAGGAAAGGGTCTGTG |  |
| *myst1* | NM_032188.2 | variant 2 | 123 | F : AGAAGCCACTGTCTGACCTG | 59 |
|  |  |  |  | R : TACTGGTCATCTGGCTGAGG |  |
| *ehmt2* | NM_025256.5 | variant 1 | 169 | F : TGCGTGCTGTTATTCCTGTC | 60 |
|  |  |  |  | R : TGATCTTCTCTGTGCGGATG |  |
| *rplp0* | NM_001002.3 | variant 2 | 223 | F : TCGACAATGGCAGCATCTAC | 60 |
|  |  |  |  | R : GCCTTGACCTTTTCAGCAAG |  |
