# Supplementary material for: Modulation of Chromatin Remodelling Induced by the Freshwater Cyanotoxin Cylindrospermopsin in Human Intestinal Caco-2 Cells
Source: PLoS One. 2014 Jun 12;9(6):e99121. doi: 10.1371/journal.pone.0099121 (PMC4055761; doi:10.1371/journal.pone.0099121)
Supplement: Table S2 — List of the 522 up-regulated and 50 down-regulated genes in differentiated Caco-2 cells after 24 hrs exposure to 1.6 µM CYN. (DOC) [file pone.0099121.s002.doc]

**Table S2.** List of the 522 up-regulated and 50 down-regulated genes in differentiated Caco-2 cells after 24 hrs exposure to 1.6 µM CYN.

| **Up-regulated genes** | **Down-regulated genes** |
| --- | --- |
| FLJ20487 | CPE |
| NSFL1C | SC4MOL |
| HBLD2 | SEC24D |
| C6orf35 | INSIG1 |
| HTATIP | AK026267 |
| TXNRD2 | SLC10A1 |
| LOC148413 | CR620977 |
| ZFAND5 | MTTP |
| MAP3K7IP1 | SCML4 |
| POLR1C | HSPA5 |
| OVCA2 | AW993939 |
| COQ9 | AQP3 |
| MRPL22 | THC2666329 |
| CRBN | CR592483 |
| QRSL1 | KLB |
| FTSJ1 | DDX46 |
| XPO6 | KCNMB4 |
| CPSF2 | AQP3 |
| C9orf156 | SCD |
| RELA | A_24_P75948 |
| CCDC58 | HMGCS1 |
| THC2645710 | TUBB |
| NHN1 | ROR1 |
| TRMT12 | TPBG |
| ZNF329 | LOC388532 |
| MGC19604 | AK022110 |
| MRPS30 | MNS1 |
| CCDC109A | MICB |
| KRT4 | AA854379 |
| MRPS18C | BM808621 |
| PRMT5 | ANXA6 |
| PFDN1 | SERPINA5 |
| THC2615064 | RAB6B |
| SLC25A26 | THC2558594 |
| MRPL39 | BG777521 |
| LOC645332 | C10orf56 |
| FARSLB | PHTF1 |
| MED6 | AP1S2 |
| UBIAD1 | DPYSL3 |
| CR613267 | THC2652817 |
| MRPS22 | CA14 |
| UMPS | GOLPH4 |
| USP4 | CACNA1E |
| FIZ1 | DDX3X |
| KLHL18 | A_24_P540560 |
| LOC144233 | HP |
| TDO2 | CFI |
| TRAPPC2L | TTN |
| UBAP1 | UTS2 |
| C1orf144 | FGA |
| COQ10B |  |
| LOC643837 |  |
| TIMM22 |  |
| RELA |  |
| ZCCHC10 |  |
| RNMTL1 |  |
| RELA |  |
| RELA |  |
| RELA |  |
| DKFZP564O0523 |  |
| N6AMT1 |  |
| MED6 |  |
| CYP1A1 |  |
| RELA |  |
| TWISTNB |  |
| C6orf55 |  |
| MRPL9 |  |
| ZC3HC1 |  |
| HSF1 |  |
| SDCCAG10 |  |
| THC2505349 |  |
| SIP1 |  |
| ALKBH8 |  |
| TMEM93 |  |
| RELA |  |
| RNF14 |  |
| MAD2L1BP |  |
| BNIP1 |  |
| DNAJC11 |  |
| MRPS10 |  |
| A_24_P272523 |  |
| RELA |  |
| DNAJA3 |  |
| DDX28 |  |
| PRR8 |  |
| CTB-1048E9.5 |  |
| C1orf149 |  |
| COPS5 |  |
| ADPRHL2 |  |
| ICT1 |  |
| ZNF313 |  |
| NGDN |  |
| AK092090 |  |
| CR593784 |  |
| TBC1D22B |  |
| POLR2L |  |
| QRSL1 |  |
| VAC14 |  |
| NFKBIB |  |
| MRPS18A |  |
| RELA |  |
| ZCCHC4 |  |
| BE646426 |  |
| NMT1 |  |
| AW804491 |  |
| PRKRIP1 |  |
| IKZF5 |  |
| FBXO28 |  |
| PRMT5 |  |
| ZNF313 |  |
| SSSCA1 |  |
| SRA1 |  |
| PDLIM3 |  |
| MKLN1 |  |
| ZNF777 |  |
| OVCA2 |  |
| PRKAA1 |  |
| FARSLB |  |
| FOXA2 |  |
| RNUXA |  |
| MGC19604 |  |
| WDR74 |  |
| MRPL50 |  |
| C14orf46 |  |
| POLR3E |  |
| GCC1 |  |
| RAB3GAP1 |  |
| C7orf36 |  |
| L3MBTL2 |  |
| KIAA0999 |  |
| DDX20 |  |
| ZNF414 |  |
| MRM1 |  |
| TMEM11 |  |
| PMS2 |  |
| PRDM11 |  |
| MGC3196 |  |
| EEF1E1 |  |
| NOB1 |  |
| MRPL42 |  |
| MGC16597 |  |
| C2orf47 |  |
| ZNF313 |  |
| BTG1 |  |
| C1orf19 |  |
| BG191465 |  |
| MRPS10 |  |
| RNF12 |  |
| NUDT16L1 |  |
| RBM18 |  |
| HBLD2 |  |
| RELA |  |
| C12orf45 |  |
| DDX47 |  |
| PRPF38A |  |
| SRFBP1 |  |
| C1orf163 |  |
| BC063022 |  |
| ZNF777 |  |
| MRPL47 |  |
| EHBP1 |  |
| TSR1 |  |
| BCL2L1 |  |
| BCL2L1 |  |
| BCL2L1 |  |
| EMG1 |  |
| PGS1 |  |
| C1orf144 |  |
| SRP68 |  |
| C19orf48 |  |
| RAB9P1 |  |
| PTPN14 |  |
| WDR74 |  |
| CPLX1 |  |
| SLC7A6OS |  |
| TWISTNB |  |
| ZBTB17 |  |
| SUV39H2 |  |
| FOXA2 |  |
| DPH2 |  |
| EMG1 |  |
| RNF25 |  |
| TP53RK |  |
| PPP1R8 |  |
| FAM86C |  |
| C4orf23 |  |
| SURF2 |  |
| PEX14 |  |
| PIGC |  |
| PYCRL |  |
| RPP40 |  |
| CTA-126B4.3 |  |
| GPR125 |  |
| FBXO28 |  |
| CR601567 |  |
| ZSWIM6 |  |
| C6orf66 |  |
| TSSC4 |  |
| THUMPD2 |  |
| TAF2 |  |
| ZNF438 |  |
| CBLL1 |  |
| ELL |  |
| RBM19 |  |
| RSF1 |  |
| CCDC55 |  |
| LOC653499 |  |
| AL117403 |  |
| METTL2B |  |
| DPH2 |  |
| UPP1 |  |
| COX10 |  |
| DENND4A |  |
| LOC348926 |  |
| CA314451 |  |
| CCDC86 |  |
| BCL2L1 |  |
| BXDC5 |  |
| ZBTB9 |  |
| ALKBH1 |  |
| ELAC1 |  |
| HEATR3 |  |
| CB305813 |  |
| TAF2 |  |
| RBM19 |  |
| THC2592963 |  |
| C6orf35 |  |
| BCL2L1 |  |
| MYNN |  |
| GPATCH4 |  |
| BXDC5 |  |
| GRWD1 |  |
| TMEM163 |  |
| NUP50 |  |
| dJ222E13.2 |  |
| NOL9 |  |
| CHAC2 |  |
| FAM83F |  |
| PAK1IP1 |  |
| MOSPD1 |  |
| A_24_P367326 |  |
| C16orf68 |  |
| BOP1 |  |
| BCL2L1 |  |
| CDC42EP3 |  |
| BCL2L1 |  |
| RNASEH1 |  |
| PDCD2L |  |
| VGLL1 |  |
| C2orf49 |  |
| SIN3B |  |
| RAB9 |  |
| CCDC55 |  |
| RABL3 |  |
| ATF4 |  |
| BC053632 |  |
| PUS1 |  |
| FLJ37953 |  |
| MMACHC |  |
| FBXL20 |  |
| CCDC43 |  |
| JTV1 |  |
| MGC14376 |  |
| MDM4 |  |
| ENST00000281228 |  |
| WDR43 |  |
| BC098428 |  |
| RRP9 |  |
| FAM86B1 |  |
| BCL2L1 |  |
| PNO1 |  |
| CARD6 |  |
| THC2677617 |  |
| BCL2L1 |  |
| PAK1IP1 |  |
| DHRS2 |  |
| FTSJ2 |  |
| BTBD14A |  |
| THC2726026 |  |
| ABTB2 |  |
| PLK3 |  |
| EPS15L1 |  |
| BC031320 |  |
| WDR42A |  |
| CCDC86 |  |
| NFKB2 |  |
| LINCR |  |
| KRT18 |  |
| IL1R2 |  |
| OXR1 |  |
| SLC20A1 |  |
| XPO6 |  |
| PRR3 |  |
| FAM46B |  |
| NFKBIB |  |
| PEO1 |  |
| ST7L |  |
| ERRFI1 |  |
| AI570240 |  |
| SLA |  |
| STK17A |  |
| MRPS10 |  |
| THC2468883 |  |
| PYCRL |  |
| RGC32 |  |
| METTL2A |  |
| FRMD5 |  |
| FOSL1 |  |
| AL359055 |  |
| LOC649514 |  |
| TMEM22 |  |
| AK055915 |  |
| MAP1D |  |
| LOC731597 |  |
| TNFRSF12A |  |
| METTL1 |  |
| L48692 |  |
| METTL1 |  |
| LOC731794 |  |
| THC2524582 |  |
| RFFL |  |
| GDF15 |  |
| RABL3 |  |
| DOK7 |  |
| LOC388743 |  |
| PPARBP |  |
| ATF3 |  |
| CYR61 |  |
| LOC391803 |  |
| UPP1 |  |
| MITF |  |
| RGC32 |  |
| TBX3 |  |
| EDN1 |  |
| FAM80B |  |
| KLF6 |  |
| ATXN7L2 |  |
| ARHGAP28 |  |
| CR608275 |  |
| CRY1 |  |
| MSI2 |  |
| BC014218 |  |
| SKAP1 |  |
| DUSP1 |  |
| GPX2 |  |
| 7A5 |  |
| PUS1 |  |
| NFKB2 |  |
| DUSP14 |  |
| A_24_P58597 |  |
| DHRS2 |  |
| RASGRP3 |  |
| A_32_P17672 |  |
| THC2656841 |  |
| A_24_P255965 |  |
| C18orf43 |  |
| FLJ45248 |  |
| A_24_P161733 |  |
| BATF |  |
| LOC391819 |  |
| CAV1 |  |
| STX1A |  |
| CXorf15 |  |
| BCL2L1 |  |
| SERTAD1 |  |
| BCL2L1 |  |
| PSCD3 |  |
| ZFP36 |  |
| CYR61 |  |
| LOC643471 |  |
| CR613654 |  |
| ANKRD1 |  |
| EGR4 |  |
| EDN1 |  |
| RASAL2 |  |
| LOC286149 |  |
| MGC4677 |  |
| LOC391271 |  |
| THC2530905 |  |
| PPFIBP1 |  |
| NFKB2 |  |
| KRTAP3-1 |  |
| ANGPT1 |  |
| ST7L |  |
| ENST00000338711 |  |
| EGR1 |  |
| RND3 |  |
| NOC3L |  |
| CCK |  |
| IER2 |  |
| JUN |  |
| ZNF593 |  |
| PHC3 |  |
| WDR48 |  |
| THC2680668 |  |
| TNFAIP3 |  |
| TFAP2C |  |
| CXCR4 |  |
| THRAP1 |  |
| LOC391827 |  |
| FOS |  |
| CABP5 |  |
| SCEL |  |
| TNS4 |  |
| RND3 |  |
| WHDC1 |  |
| IL1R2 |  |
| JUN |  |
| HIST1H4H |  |
| STX1A |  |
| LOC642448 |  |
| GADD45B |  |
| FAM86A |  |
| C14orf138 |  |
| LOC401317 |  |
| XAB2 |  |
| IFRD1 |  |
| NR4A3 |  |
| LOC644030 |  |
| GADD45B |  |
| C2orf37 |  |
| ATXN7L4 |  |
| NOC3L |  |
| ARL14 |  |
| AK000257 |  |
| RND3 |  |
| EDN1 |  |
| PLK2 |  |
| CXCR4 |  |
| RND3 |  |
| JUN |  |
| IFRD1 |  |
| KLF6 |  |
| EDN1 |  |
| ZMYM5 |  |
| A_24_P792988 |  |
| RND3 |  |
| RND3 |  |
| JUN |  |
| JUN |  |
| DLX2 |  |
| JUN |  |
| TMCO7 |  |
| JUNB |  |
| DOCK5 |  |
| CAV1 |  |
| GCNT3 |  |
| LOC132391 |  |
| MRPL1 |  |
| SLC7A6 |  |
| C8orf75 |  |
| FHL2 |  |
| JUN |  |
| JUN |  |
| LOC391179 |  |
| AK023018 |  |
| JUN |  |
| CB529149 |  |
| LOC387763 |  |
| RND3 |  |
| HIST2H4B |  |
| SOCS4 |  |
| NR1D1 |  |
| DUSP16 |  |
| A_23_P251002 |  |
| CXCR4 |  |
| LOC139060 |  |
| CXCR4 |  |
| FOSB |  |
| NOC3L |  |
| ENST00000308604 |  |
| NR1D1 |  |
| CXCR4 |  |
| LOC651439 |  |
| RND3 |  |
| PGS1 |  |
| S100A2 |  |
| DUSP4 |  |
| MAP3K14 |  |
| CYLD |  |
| A_24_P110521 |  |
| SH2D3A |  |
| GPRC5A |  |
| CXCR4 |  |
| EDN1 |  |
| RND3 |  |
| A_23_P15233 |  |
| PLEKHG1 |  |
| AA747799 |  |
| RELB |  |
| PRICKLE1 |  |
| LHFP |  |
| NR4A1 |  |
| JUN |  |
| EDN1 |  |
| BM474343 |  |
| NFKB2 |  |
| SAMD4A |  |
| HIST1H4F |  |
| CXCR4 |  |
| PHLDA1 |  |
| NOC3L |  |
| SLC25A25 |  |
| C7orf40 |  |
| C6orf152 |  |
| DUSP5 |  |
| NEDD9 |  |
| ZNF461 |  |
| THC2709754 |  |
| THC2550463 |  |
| CXCR4 |  |
| ChGn |  |
| CXCR4 |  |
| TFE3 |  |
| NOSTRIN |  |
| FAM122A |  |
| MAFF |  |
| ENST00000366874 |  |
| PHLDA2 |  |
| AW979273 |  |
| EDN1 |  |
| EDN1 |  |

The differentially-expressed genes were selected at *P* < 0.05 (Student t-test) and with a fold change (FC) greater than 2 (for “up-regulated genes”), or less than 0.5 (for “down-regulated genes”).
