# Supplementary material for: Modulation of Chromatin Remodelling Induced by the Freshwater Cyanotoxin Cylindrospermopsin in Human Intestinal Caco-2 Cells
Source: PLoS One. 2014 Jun 12;9(6):e99121. doi: 10.1371/journal.pone.0099121 (PMC4055761; doi:10.1371/journal.pone.0099121)
Supplement: Table S3 — Cellular components and molecular functions for up-regulated genes in differentiated Caco-2 cells after 24 hrs exposure to 1.6 µM CYN. (DOC) [file pone.0099121.s003.doc]

**Table S3**. Cellular components and molecular functions for up-regulated genes in differentiated Caco-2 cells after 24 hrs exposure to 1.6 µM CYN.

| **GO category** | **GO numeric identifier** | **GO term** | **enrichment score** | **FDR score** |
| --- | --- | --- | --- | --- |
| Cellular component | 0005730 | nucleolus | 2.63 | 0 |
| Cellular component | 0031981 | nuclear lumen | 1.83 | 0 |
| Cellular component | 0044428 | nuclear part | 1.69 | 0 |
| Cellular component | 0070013 | intracellular organelle lumen | 1.79 | 0 |
| Cellular component | 0043233 | organelle lumen | 1.76 | 0 |
| Cellular component | 0031974 | membrane-enclosed lumen | 1.73 | 0 |
| Cellular component | 0005763 | mitochondrial small ribosomal subunit | 8.93 | 0.0186 |
| Cellular component | 0005761 | mitochondrial ribosome | 6.70 | 0.0008 |
| Cellular component | 0000314 | organellar small ribosomal subunit | 8.93 | 0.0186 |
| Cellular component | 0000313 | organellar ribosome | 6.70 | 0.0008 |
| Cellular component | 0005840 | ribosome | 2.99 | 0.0086 |
| Cellular component | 0030529 | ribonucleoprotein complex | 2.26 | 0.0031 |
| Molecular function | 0003713 | transcription coactivator activity | 2.76 | 0.0300 |
| Molecular function | 0003714 | transcription corepressor activity | 3.29 | 0.0273 |
| Molecular function | 0003712 | transcription cofactor activity | 2.96 | 0 |
| Molecular function | 0008134 | transcription factor binding | 2.46 | 0.0013 |
| Molecular function | 0017017 | MAP kinase tyrosine/serine/threonin phosphatase activity | 17.64 | 0 |
| Molecular function | 0033549 | MAP kinase phosphatase activity | 17.64 | 0 |
| Molecular function | 0008138 | protein tyrosine/serine/threonin phosphatase activity | 5.71 | 0.0357 |
| Molecular function | 0008173 | RNA methyltransferase activity | 8.17 | 0.0292 |
| Molecular function | 0008168 | methyltransferase activity | 4.13 | 0 |
| Molecular function | 0016741 | transferase activity transferring one-carbon groups | 4.07 | 0 |
| Molecular function | 0003735 | structural consistuent of ribosome | 2.85 | 0.0340 |

The 522 genes showing differential up-regulation were annotated with 12 significant cellular components and 11 significant molecular functions using GoMiner software. The GO terms had an enrichment score greater than 1.5, and a false discovery rate (FDR) score less than 0.05.
