# Supplementary material for: Modulation of Chromatin Remodelling Induced by the Freshwater Cyanotoxin Cylindrospermopsin in Human Intestinal Caco-2 Cells
Source: PLoS One. 2014 Jun 12;9(6):e99121. doi: 10.1371/journal.pone.0099121 (PMC4055761; doi:10.1371/journal.pone.0099121)
Supplement: Table S4 — Categories and biological functions for differentially regulated genes in differentiated Caco-2 cells after 24 hrs exposure to 1.6 µM CYN. (DOC) [file pone.0099121.s004.doc]

**Table S4**. Categories and biological functions for differentially regulated genes in differentiated Caco-2 cells after 24 hrs exposure to 1.6 µM CYN.

| **Category** | **Biological function** | **Number of associated molecules** | ***P*-value** |
| --- | --- | --- | --- |
| DNA Replication, Recombination, and Repair | DNA damage response of cells | 12 | 0.00008 |
| DNA Replication, Recombination, and Repair | Repair of DNA | 15 | 0.00010 |
| DNA Replication, Recombination, and Repair | Modification of DNA | 16 | 0.00114 |
| Gene Expression | Transcription | 67 | 0.00035 |
| Gene Expression | Transcription of DNA endogenous promoter | 36 | 0.00098 |
| RNA Post-Transcriptional Modification | Modification of RNA | 14 | 0.00030 |
| RNA Post-Transcriptional Modification | Processing of RNA | 11 | 0.00272 |

The 2911 genes showing differential regulation were shared out within 3 categories including 7 significant biological functions using IPA software. The biological functions had at least 10 molecules involved and a *P*-value of less than 0.01.
